# Trophoblast Migration with Different Oxygen Levels in a Gel-Patterned Microfluidic System

**DOI:** 10.3390/mi13122216

**Published:** 2022-12-14

**Authors:** Gun Ko, Tae-Joon Jeon, Sun Min Kim

**Affiliations:** 1 Department of Biological Sciences and Bioengineering, Inha University, 100 Inha-ro, Michuhol-gu, Incheon 22212, Republic of Korea; 2 Department of Biological Engineering, Inha University, 100 Inha-ro, Michuhol-gu, Incheon 22212, Republic of Korea; 3 Department of Mechanical Engineering, Inha University, 100 Inha-ro, Michuhol-gu, Incheon 22212, Republic of Korea

**Keywords:** placenta, trophoblast invasion, hypoxia, gel-patterned system, microfluidic system

## Abstract

In the placenta, substances such as nutrients, oxygen, and by-products are exchanged between the mother and the fetus, and the proper formation of the placenta determines the success of pregnancy, including the growth of the fetus. Preeclampsia is an obstetric disease in which the incomplete formation of the placenta occurs, which is known to occur when there is an abnormality in the invasion of trophoblast cells. The invasion of trophoblast cells is controlled by oxygen concentration, and HIF-1α changes according to oxygen concentration, showing a difference in cell mobility. MMP-2 and MMP-9 are observed to be high in the endometrium involved in trophoblast invasion, and the expression is regulated according to the oxygen concentration. In this experiment, cell culture was conducted using a gel-patterned system with a hypoxic chamber. Before the chip experiment, the difference in the expression of MMP-2 and MMP-9 according to the oxygen concentration was confirmed using a hypoxia chamber. After that, trophoblast cells (HTR8/SVneo) and endothelial cells (HUVECs) were separated and cultured through a physical barrier through a hydrogel on a microfluidic chip. Cells were cultured in a hypoxic chamber under controlled oxygen levels. It was confirmed that the mobility of trophoblast cells in culture on the chip was upregulated in a hypoxic environment through oxygen control. This suggests that the formation of a hypoxic environment in the endometrium where the invasion of trophoblast cells occurs plays a role in increasing cell mobility.

## 1. Introduction

The placenta is a temporary organ that exchanges substances between the mother and the fetus during pregnancy, and the proper formation of the placenta determines the success of pregnancy [1]. Placentation begins with the trophoblast invasion, in which the differentiated trophoblast migrates into the endometrium and invades the material spatial artery [2]. In the early stage of trophoblast invasion, a low oxygen partial pressure of 1–3% O_2_ is maintained in the early stages of pregnancy, and then the normal oxygen partial pressure is restored to a range of 5–8% O_2_ due to spiral artery expansion [3]. However, in the case of preeclampsia, the spiral artery does not expand properly, and a hypoxic environment is continuously maintained [4]. This inadequate development occurs in preeclampsia, leading to high blood pressure, proteinuria, and edema [5,6,7]. Therefore, it is important to study the trophoblast invasion mechanism under a hypoxic environment. The hypoxic environment is a major factor that increases trophoblast invasion [8,9]. Hypoxia-induced factor-1 alpha (HIF-1α) expression in the endometrium is clinically observed [10]. Oxygen level affects the degree of degradation of HIF-1α, which promotes gene expression by binding to the hypoxia response element (HRE) [11,12]. It also affects the matrix metalloproteinase (MMP) expression, which is involved in cell migration via the degradation of the extracellular matrix [13]. MMP is an essential factor that decomposes substrates for each type and regulates cell mobility [14]. It is known that the expression of MMP-2 and MMP-9 in the MMP subfamily is responsible for the mobility of trophoblasts [15], and it has been confirmed in many studies that a hypoxic environment increases the expression of MMP-2 and MMP-9 in trophoblasts [16].

In trophoblast invasion studies, the traditional 2D culture has limitations in measuring the cell migration distance and direction with co-culture [17]. Recent research is being conducted using microfluidic chips for cell research to solve this problem [18,19,20]. A microfluidic chip can separate a cell culture area through various hydrogels. It is possible to analyze interaction with the substance diffusion across the hydrogel structure, which has a porous structure. It can be set to analyze cell migration distance and direction. In addition, Polydimethylsiloxane (PDMS) used in microfluidic chips is a famous material with high gas permeability, and there is an advantage that oxygen can be controlled for cellular respiration.

**Figure 1:**
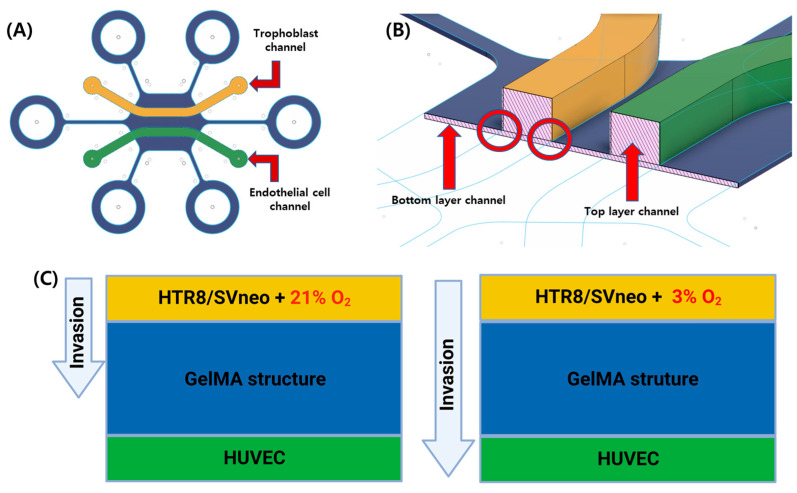
Schematic figure of the gel-patterning microfluidic chip. (**A**) The cell culture areas are separated by GelMA structure. Trophoblast channel (yellow, HTR8/SVneo); endothelial cell channel (green, HUVECs). (**B**) The gel-patterning chip consists of a double layer of PDMS with different heights. Bottom (50 μm); top (400 μm). This height difference means the GelMA solution can only pattern according to the non-over-lapping area. (**C**) Comparison of trophoblast (HTR8/SVneo) migration with normoxic (21% O_2_) and hypoxic (3% O_2_) conditions.

This experiment was designed to experiment on the analysis of changes in cell mobility through oxygen control. Before proceeding with the experiment on the chip, the change in mRNA expression in cells through oxygen regulation was analyzed. The chip was designed to check cell movement due to the difference in oxygen concentration. The chip was fabricated via gel patterning through the height difference (Figure 1). Generally, microfluidic chips are manufactured using a natural hydrogel with low stiffness [21]. In our chip fabrication, the microfluidic chip was manufactured through gelatin methacrylate (GelMA), paying attention to the fact that the high stiffness [22,23,24], like that of the tissue, also affects cell movement. After manufacturing the chip, trophoblast migration was observed through cell culture for each channel. At this time, the oxygen concentration was controlled through the hypoxic chamber.

## 2. Materials and Methods

### 2.1. Cell Culture

Cells were cultured in T-75 flasks before culturing in microfluidic chips. A human trophoblast cell line (HTR8/SVneo; donated to Cha medical university) was incubated in RPMI1640 (Cytiva, SH30255.01, Longan, UT, USA), mixed with 5% fetal bovine serum (FBS; Gibco, 10082147, Thermo Fisher Scientific, Waltham, MA, USA) and 1% penicillin/streptomycin (P/S; Gibco, 15140122).

Human Umbilical Vein Endothelial cells (HUVECs, ScienCell Research Laboratories, 8000, San Diego, CA, USA) were cultured using endothelial cell media (ScienCell Research Laboratories, 1001, USA) mixed with endothelial cell growth supplement (ECGS, ScienCell Research Laboratories, 1052), 5% FBS (ScienCell Research Laboratories, 0025, USA), and 1% P/S (ScienCell Research Laboratories, 0503, USA). Cells were cultured at 37 °C and 5% CO_2_ in an incubator.

### 2.2. Gelatin Methacrylate Solution

The gelatin–methacrylate (GelMA) used in the experiment was produced according to the company’s manual. The photo-initiator (PI), lithium phenyl-2,4,6-trimethyl-benzoyl phosphate (LAP; CELLINK, CKVLLP00010001, Gothenburg, Sweden), was dissolved in Reconstitution Agent P (CELLINK, IKR300000050, Sweden) at a concentration of 0.1%. The lyophilized GelMA (CELLINK, VL3500000502, Sweden) was mixed with a PI solution at 8 *w*/*w*% via a hotplate at 50 °C with a magnetic stirrer. Then, aliquoted GelMA solution was stored in 4 °C refrigerators before use.

### 2.3. Device Fabrication

The chip was manufactured through a photolithography process using the SU8 photoresist series (Kayaku Advanced Materials, Westborough, MA, USA). To proceed with gel patterning, the heights of the chips were made to differ. SU8-2050 was used on a 4-inch silicon wafer with 50 µm height for the bottom layer mold. SU8-2150 was used with 400 µm for the top layer mold. Each photoresist was spread using a spin coater (POLOS,150i) in each height condition. Soft baking, UV exposure, hard baking, and development were performed according to manual requirements with each height condition. They were stored overnight in a 68 °C oven for stabilization.

Polydimethylsiloxane (PDMS; Dow corning, Midland, MI, USA, SYLGARD 184) and curing agent were mixed in a ratio of 10:1 *w*/*w* and degassed via a vacuum pump for soft lithography using a chip mold. The PDMS mixture was poured into the chip mold and reacted for curing in 68 °C ovens for 4 h. Cured PDMS was trimmed with surgical mesh and bio punching (1 mm). For sterilization, the PDMS chip underwent UV exposure for a least 30 min on a clean bench.

### 2.4. Gel Patterning and Cell Seeding

The bottom layer of PDMS was attached to the slide glass after the plasma treatment (60 W, 1 min). Then, the top layer was attached to the bottom layer according to the align key after the same plasma treatment with the bottom layer. The GelMA solution was injected into the gel channel inlet to perform gel patterning. The UV exposure treatment was performed for 5 min at room temperature. To improve cell adhesion, 50 ng/mL of fibronectin (Sigma-Aldrich, Saint Louis, MO, USA. F1141) was injected into a cell channel and reacted at 37 °C for 2 h in a humid chamber, being washed twice with DPBS.

The cultured cells in a T-75 flask were detached using 0.25% Trypsin-EDTA (Gibco, 15400), and the cell pellet was diluted at a concentration of 10^7^ cells/mL. The diluted cell solution was injected into each cell channel. After incubation for 2 h in an incubator, the medium replacement was performed to confirm the adhesion of cells and remove non-attached cells. The chips were cultured in an incubator (21% O_2_) and a hypoxia chamber (3% O_2_). Media were replaced every two days.

### 2.5. Molecular Diffusion Analysis

The degree of diffusion through fluorescence was measured to check the possibility of cell interaction through the GelMA structure. Without cell seeding, 1.0 mg/mL of 40 kDa of FITC-dextran (Sigma-Aldrich, FD-40) was injected into the trophoblast channel. Fluorescence images of trophoblast and endothelial cell channels were obtained at 2 h intervals with a time-lip using a fluorescence microscope (Etaluma Inc., Carlsbad, CA, USA, LS620). ImageJ was used to calculate fluorescence intensity.

### 2.6. Cell Tracker Staining

Cell tracker staining was performed before the cell seeding process. The HUVECs were dyed using 5 ug/mL of CellTracker™ Red CMTPX Dye (Invitrogen, Carlsbad, CA, USA C34552), and HTR8/SVneo were dyed using 5 ug/mL of CellTracker™ Green CMFDA Dye (Invitrogen, C7025). The cell tracker was diluted using a fresh medium suitable for each cell. After replacing the media with a working solution, these were incubated at 37 °C for 30 min, then washed twice with DBPS. Dyed cells were used in the cell seeding process. Fluorescence images were captured using a fluorescence microscope (Etaluma Inc., LS620).

### 2.7. Quantitative Real-Time PCR (qRT-PCR)

Quantitative RT-PCR was performed to measure the MMP-2 and MMP-9 mRNA expression in trophoblasts according to oxygen level (21%, 8%, and 3% O_2_). The HTR8/SVneo were seeded in a 6-well plate with 3 × 10^5^ cells per well and incubated for 24 h in an incubator (37 °C and 5% CO_2_). After that, they were replaced with a fresh medium and incubated for 24 h under different oxygen conditions. In the 21% O_2_ condition, they were incubated in the incubator. In other oxygen conditions (8% O_2_ and 3% O_2_), they were incubated in a hypoxic chamber (BioSpherix, Parish, NY, USA. ProOx110).

After that, RNA extraction was performed using the RNeasy Mini Kit (QIAGEN, Valencia, CA, Spain. 74104). This was carried out according to the manufacturer’s manual. After RNA extraction, the concentration of RNA was measured using nanodrop. The RNA sample was diluted to 100 ng/uL using RNA-free water. Then, cDNA was prepared using Primescript™ RT Master Mix (Takara Bio Inc., RR036, Kusatsu, Japan,). RT-PCR was then performed in triplicate using TB Green^®®^ Premix Ex Taq™ II (Takara Bio Inc., RR820).

The real-time PCR was carried out using an RT-PCR system (Bio-Rad Laboratories, Hercules, CA, USA, CFX Opus Real-Time PCR Systems). The following thermal cycle condition was used (Step 1. Initial denaturation 95 °C for 30 s; Step 2. PCR: followed by 40 cycles of 95 °C for 5 s/60 °C for 30 s; Step 3. Melting curve). The following primer pairs were used: Beta-actin forward (5-CAC CAT TGG CAA TGA GCG GTT C-3), Beta-actin reverse (5-AGG TCT TTG CGG ATG TCC ACG T-3), MMP-2 forward (5′-AGC GAG TGG ATG CCG CCT TTA A-3′), MMP-2 reverse (5-CAT TCC AGG CAT CTG CGA TGA G-3), MMP-9 forward (5-GCC ACT ACT GTG CCT TTG AGT C-3) and MMP-9 reverse (5-CCC TCA GAG AAT CGC CAG TAC T-3). The mRNA levels of MMP-2 and MMP-9 were normalized with the reference gene of beta-actin. Data were plotted using the Origin program (Origin lab Corp., Northampton, MA, USA).

## 3. Results and Discussion

### 3.1. Gel Patterning in a Microfluidic Chip

In order to perform co-culture in microfluidic chips, various methods are used to form a physical barrier for cell culture area separation. Cells are separated by inserting a porous membrane or hydrogel to form a physical barrier inside the chip. Among the hydrogel methods, gel patterning uses the hydrophilicity and low height of the surface rather than injecting liquid pressure to pattern a physical barrier via capillary action.

**Figure 2:**
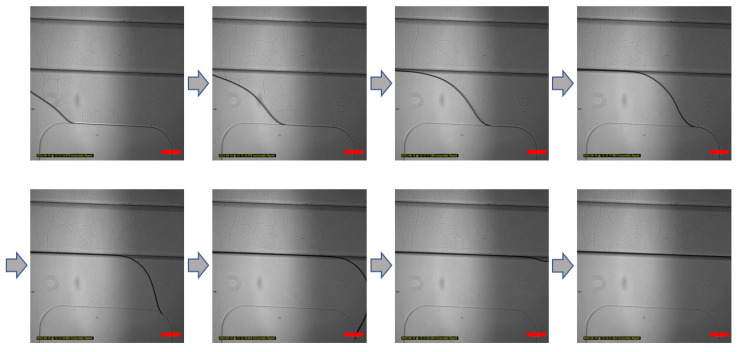
Gel patterning of GelMA solution in the microfluidic chip. GelMA solution patterning image according to time. The GelMA solution does not leak into their overlap areas. According to the boundary of only the bottom layer channel, the GelMA solution is moved by capillary force. Scale bar: 200 μm.

In the chip used in this experiment, the transfer process of the GelMA solution without external pressure on the hydrophilic surface through plasma treatment is shown (Figure 2). Bottom layer to top layer bonding enables surfaces to bond through oxygen plasma. PDMS undergoes surface modification with hydrophilicity due to plasma treatment, and the formation of such a hydrophilic surface enables capillary action by a small height. The overlapping part of the bottom and top layers forms the height difference with bottom layer. It can be observed that the solution only moves in the non-overlapping bottom channel.

The gel patterning method using the capillary phenomenon can reduce the manufacturing difficulty of handling differences in chip manufacturing through solution injection if hydrophilic modification is achieved through appropriate surface treatment.

### 3.2. Cell Separation and Molecular Diffusion of GelMA Structure

**Figure 3:**
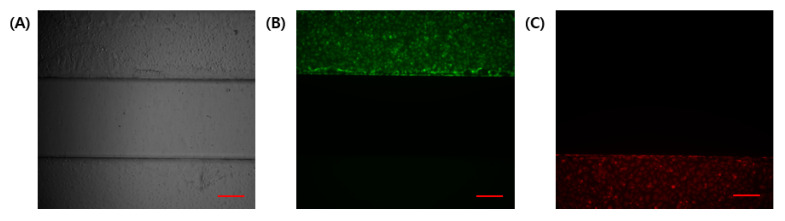
Determination of the independence of the channel during the cell seeding process. (**A**) Bright-field image. (**B**) HTR8/SVneo with cell tracker green. (**C**) HUVEC with cell tracker red. Scale bar (red): 200 μm.

In a co-culture using different cell lines in microfluidic chips, independent channels are required to be separated using physical barriers. In this experiment, cell attachment to the coating was enabled using suction. GelMA has high physiological properties and is easy to handle without gel collapse. The compressive modulus of GelMA gel from the solution is about 20~30 kPa [24]. The separability between channels was confirmed using a fluorescence microscope (Figure 3). Fluorescent signals in the GelMA structure were not identified. Hence, no cells entered the gel structure during the cell seeding process.

**Figure 4:**
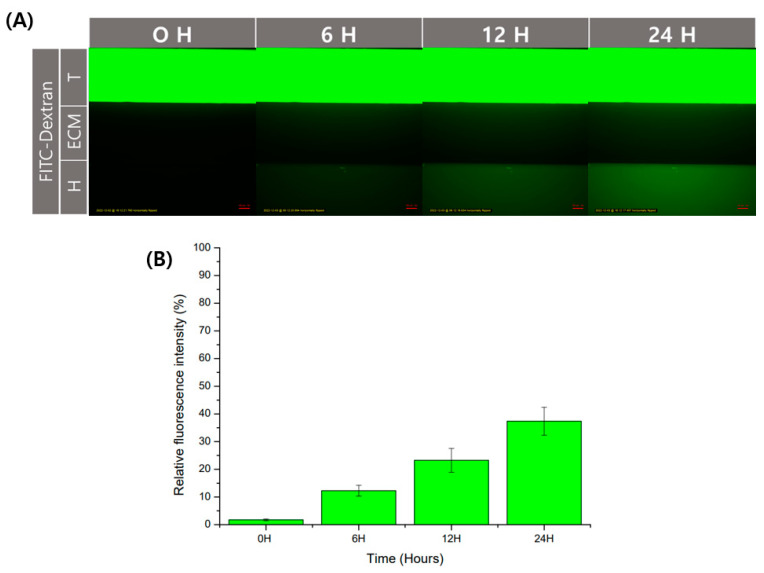
Verification of diffusion of substances in GelMA structure. The diffusion of the substance in the structure using GelMA was confirmed using an FITC-dextran. (**A**) Fluorescence image according to time. Scale bar: 100 μm. (**B**) Relative fluorescence intensity was calculated based on the trophoblast channel. Data are expressed with mean ± standard deviations (*n* = 3).

Physical barriers using hydrogels have the advantage of being able to diffuse. Diffusion allows for cell interaction by transferring substances secreted by cells. In order to check the degree of diffusion in the GelMA structure used in this experiment, we used fluorescent molecules. As a fluorescent substance, the experiment was conducted using FITC-dextran, often used in diffusion experiments. It was observed that FITC-dextran with a size of 40 kDa was injected into the trophoblast channel and then diffused over time to the endothelial channel. (Figure 4A). After that, as a result of analyzing the fluorescence intensity through ImageJ, the fluorescence intensity degree in the endothelial channel was shown to increase over time (Figure 4B). It could be used without any problems in the transfer of substances for the interaction of cells between channels.

### 3.3. Hypoxia Promote MMP-2 and MMP-9 mRNA Expression in HTR8/SVneo

It is known that a hypoxic environment is crucial in regulating cell migration in trophoblast invasion. MMP-2 and MMP-9 are the most distinctly expressed proteins in the endometrium where trophoblast invasion occurs. The expression of MMP-2 and MMP-9 is regulated by oxygen level. MMP is a family of proteins involved in the degradation of the extracellular matrix, and MMP-2 and MMP-9 are classified in gelatinase, which can degrade gelatin and collagen. 

**Figure 5:**
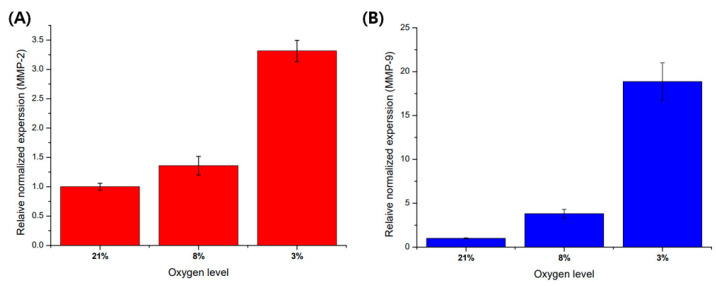
Comparison of MMP-2 and MMP-9 mRNA expression with oxygen levels in trophoblast (HTR8/SVneo). (**A**) MMP-2 mRNA expression in different oxygen levels. (**B**) MMP-9 mRNA expression in different oxygen levels. These mRNA expressions are normalized with 21% O_2_ condition for control using beta action for the reference gene. Data are expressed with mean ± standard deviations (*n* = 3).

Before cell culture in a microfluidic chip, a pre-experiment was performed on a 6-well plate to analyze the regulation of gene expression according to oxygen level. As a result of experimenting with an oxygen gradient using a hypoxic chamber with an adjustable oxygen level, as the oxygen level decreased, the mRNA expression of MMP-2 and MMP-9 was affected by the oxygen levels (Figure 5). The expression of MMP-9 mRNA showed a higher trend of increase in the hypoxic environment compared to other oxygen concentration conditions.

### 3.4. Comparison of Trophoblast Cell Migration with Different Oxygen Levels

Chip cultivation was performed in a hypoxia chamber to show cell migration at different oxygen levels. The chip design used a top layer with an interval of 800 μm. Cell seeding was carried out for each channel, washing non-adherent cells by washing culture media. The first media washing timing was set for the day 0 condition. Chip culture was used to perform static culture using the yellow tip for a reservoir. Bright-field images were acquired while changing the medium every 48 h.

**Figure 6:**
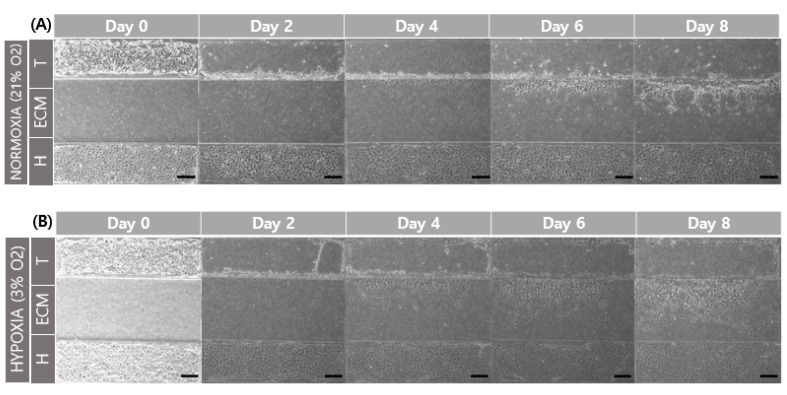

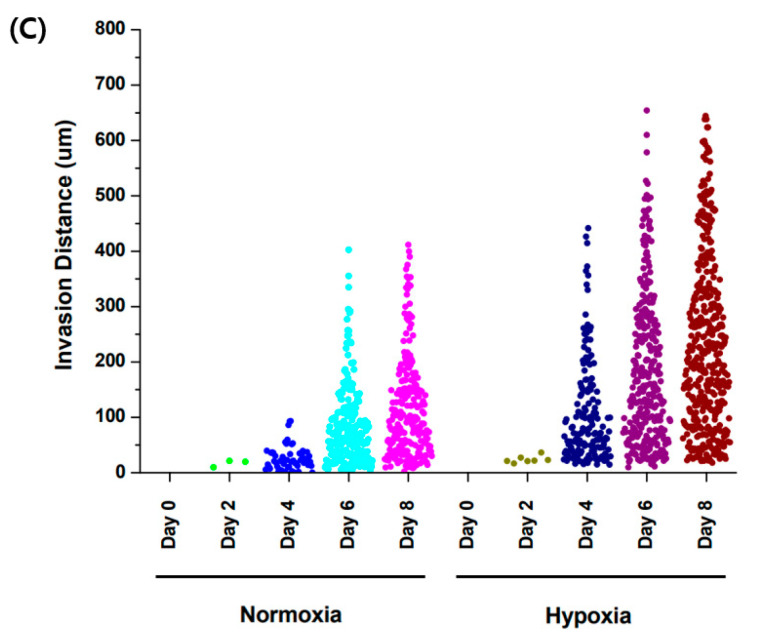
Comparison of trophoblast (HTR8/SVneo) migration in different oxygen levels. (**A**) Co-culture with HUVEC in normoxia (21% O_2_). (**B**) Co-culture with HUVEC in hypoxia (3% O_2_). Scale bar = 200 μm. (**C**) Migration distance of trophoblast in different oxygen levels.

When comparing the normoxic (21% O_2_) and hypoxic (3% O_2_) environments, and a comparison of cell migration at day 6 and 8 showed that hypoxia promoted cell migration in the GelMA structure (Figure 6). This result is related to increased MMP-2 and MMP-9 gene expression according to oxygen concentration. The structure of GelMA shared the same amino acid sequence in collagen. The increase in the gelatinases of MMP-2 and MMP-9 also affected gelatin degradation for trophoblast invasion.

## 4. Conclusions

In this study, we analyzed the migration of trophoblast cells through a gel patterning chip. In addition, changes in mobility were analyzed by mimicking the oxygen environment of the endometrium in a hypoxia chamber. In the cell migration evaluation, a 3D structure was fabricated using GelMA, which had a higher stiffness than the natural hydrogel, thereby limiting indiscriminate cell movement due to the low stiffness of the natural hydrogel. This differs because it aims to only migrate cells affected by mobility in terms of mimicry. The mRNA expression of MMP-2 and MMP-9, migration-related proteins through oxygen level control, was confirmed, and the expression level increased in a hypoxic environment. Subsequently, the incubation on the chip showed cell migration was regulated by oxygen concentration. According to the results of this experiment, the existence of a hypoxic environment asserted in cell migration in trophoblast invasion means that it is a factor that enhances trophoblast cell invasion.

## Data Availability

Not applicable.
